# Psychosocial Factors and Glycemic Control in Young Adults With Youth-Onset Type 2 Diabetes

**DOI:** 10.1001/jamanetworkopen.2024.5620

**Published:** 2024-04-08

**Authors:** Paula M. Trief, Hui Wen, Brian Burke, Diane Uschner, Barbara J. Anderson, Xun Liu, Jane Bulger, Ruth S. Weinstock

**Affiliations:** 1 Department of Psychiatry and Behavioral Sciences, State University of New York Upstate Medical University, Syracuse; 2 Biostatistics Center, George Washington University, Rockville, Maryland; 3 Department of Pediatrics-Psychology, Baylor College of Medicine, Houston, Texas; 4 Department of Medicine, State University of New York Upstate Medical University, Syracuse

## Abstract

**Importance:**

Youth-onset type 2 diabetes is associated with poor glycemic control and early onset of complications. Identification of psychosocial factors associated with poor glycemic control is needed to inform efficacious interventions.

**Objective:**

To identify psychosocial factors associated with glycated hemoglobin (HbA_1c_) levels in young adults with youth-onset type 2 diabetes.

**Design, Setting, and Participants:**

For the *i*Count cohort study, HbA_1c_ levels were measured twice (at baseline [T1] and at 1 year [T2]) during the last years (2017-2019) of the observational phase of the multicenter Treatment Options for Type 2 Diabetes in Adolescents and Youth (TODAY2) study. Participants were young adults who had been diagnosed with type 2 diabetes during childhood or adolescence. Data were analyzed from December 2021 to September 2023.

**Main Outcomes and Measures:**

Glycemic control was examined categorically (high [≥8.0%] vs low [<8.0%] HbA_1c_), continuously (HbA_1c_ level), and over time (change in HbA_1c_: decreased ≥0.5%, remained stable, or increased ≥0.5%). Psychosocial measures included beliefs about medicines, depression and anxiety symptoms, diabetes distress, diabetes self-efficacy, self-management support, and unmet material needs. Multivariable logistic and linear regression models evaluated the association of each psychosocial factor with the probability of T2 HbA_1c_ of 8.0% or greater, T2 HbA_1c_ level, and change in HbA_1c_.

**Results:**

Of the 411 TODAY2 participants approached, 381 enrolled in the *i*Count study, and 348 with T1 and T2 HbA_1c_ data comprised the analysis group. The 348 participants had a mean (SD) age of 26.1 (2.5) years and a mean (SD) HbA_1c_ of 9.4% (2.8%). Most participants (229 [65.8%]) were women. In adjusted multivariable regressions, greater beliefs that diabetes medicines are necessary (odds ratio [OR], 1.19 [95% CI, 1.03-1.37]; *P* = .02), concerns about medicines (OR, 1.20 [95% CI, 1.00-1.45]; *P* = .049), diabetes distress (OR, 1.08 [95% CI, 1.02-1.15]; *P* = .006), and high distress (OR, 2.18 [95% CI, 1.15-4.13]; *P* = .02) increased the odds of high HbA_1c_ at T2. Greater support (OR, 0.67 [95% CI, 0.46-0.97]; *P* = .04) and diabetes self-efficacy (OR, 0.91 [95% CI, 0.84-0.99]; *P* = .02) decreased the odds of high HbA_1c_ at T2. Diabetes distress was associated with higher HbA_1c_ level at T2 (coefficient, 0.08 [95% CI, 0.02-0.13]; *P* = .01). Beliefs that diabetes medicines are necessary (OR, 1.20 [95% CI, 1.03-1.39]; *P* = .02) and concerns about medicines (OR, 1.22 [95% CI, 1.00-1.47]; *P* = .048) increased the odds of an HbA_1c_ decrease of at least 0.5% over 1 year.

**Conclusions and Relevance:**

In this cohort study of young adults with youth-onset type 2 diabetes, beliefs about medicines, high diabetes distress, low diabetes self-efficacy, and self-management support were associated with high HbA_1c_ over time. Future research should assess whether interventions that address these factors result in improved glycemic control in this at-risk group.

## Key Points

**Question:**

What psychosocial factors are associated with glycated hemoglobin (HbA_1c_) levels in young adults with youth-onset type 2 diabetes?

**Findings:**

In this cohort study of 348 participants, diabetes distress, beliefs that medicines are necessary, and concerns about medicines were associated with higher odds of high HbA_1c_ (≥8.0%) 1 year later; these beliefs were also associated with an HbA_1c_ decrease of at least 0.5%. Self-management support and self-efficacy were associated with lower odds of high HbA_1c_, whereas diabetes distress was associated with higher HbA_1c_ level.

**Meaning:**

These findings suggest that high HbA_1c_ is associated with psychosocial factors that may be targets for future interventions to improve glycemic control in young adults with youth-onset type 2 diabetes.

## Teaser

This cohort study examines psychosocial factors associated with glycated hemoglobin levels in young adults with youth-onset type 2 diabetes.

## Introduction

Outcomes in type 2 diabetes are heavily determined by self-management behaviors that often affect glycemic control.^1^ Several studies seek to identify psychosocial factors associated with glycemic control, commonly defined by hemoglobin A_1c_ (HbA_1c_) levels. Psychosocial factors studied include depressive symptoms,^2^ anxiety symptoms,^3^ diabetes distress,^4^ social support,^5^ self-efficacy,^6^ and social determinants of health.^7^ Significant factors might serve as targets of behavioral interventions to improve outcomes. However, the evidence of associations between these factors and HbA_1c_ is mixed; the available studies were mostly cross-sectional, and participants were middle-aged to older adults with adult-onset type 2 diabetes (diagnosed after age 18 years).

There has been a marked increase in the incidence and prevalence of youth-onset type 2 diabetes (diagnosed before age 18 years).^8^ Compared with adult-onset diabetes, youth-onset diabetes is associated with poorer health outcomes, including earlier onset and severity of diabetes-related complications.^9,10,11^ Developmental, social, and emotional challenges among this cohort make it difficult to generalize from existing research on older adults. The Treatment Options for Type 2 Diabetes in Adolescents and Youth (TODAY) study recruited a large, diverse sample of 699 children and adolescents with youth-onset type 2 diabetes to participate in a randomized clinical trial to evaluate 3 treatment regimens.^12^ Following the TODAY intervention study, 572 participants enrolled in the TODAY2 observational follow-up study of a unique cohort (ie, young adults with youth-onset type 2 diabetes). Looking at one psychosocial factor (depression) and HbA_1c_, 14.8% of TODAY youth participants reported clinically significant depression symptoms, but symptoms were not associated with HbA_1c_.^13^ Over the last 6 years of the TODAY2 study when participants moved into adulthood, the percentage of participants with significant depression symptoms increased (from 14.0% to 19.2%) and symptoms were associated with higher HbA_1c_, hypertension, and progression of retinopathy.^14^

There are gaps in the literature. First, there are no studies of the association of psychosocial factors with HbA_1c_ in young adults with youth-onset type 2 diabetes; existing data are from middle-aged to older adults with adult-onset type 2 diabetes and may not generalize. Second, previous studies have been cross-sectional, thus longitudinal data are needed. Finally, unstudied psychosocial factors (eg, beliefs about medicines) should be included. To address these gaps, the present study used data from the TODAY2 *i*Count study (an ancillary study of medication adherence and health care usage among the TODAY2 cohort), including repeated measures of HbA_1c_ and selected psychosocial factors. The primary *i*Count results were published previously.^15,16,17^ The aim of these preplanned secondary analyses was to identify psychosocial factors associated with HbA_1c_ over time in this cohort.

## Methods

### TODAY, TODAY2, and *i*Count

The TODAY, TODAY2, and *i*Count studies were approved by institutional review boards at 15 participating centers. Informed consent (and assent for children and adolescents when applicable) was obtained from all participants. This cohort study followed the Strengthening the Reporting of Observational Studies in Epidemiology (STROBE) reporting guideline.

For the TODAY study, children and adolescents (aged 10 to <18 years) were recruited at 15 US centers. They had recently (within 2 years) received a type 2 diabetes diagnosis (using 2004 American Diabetes Association criteria^18^) and had a body mass index (BMI; calculated as weight in kilograms divided by height in meters squared) at or above the 85th percentile, negative islet cell autoantibodies, and fasting C-peptide greater than 0.6 ng/mL (to convert to nmol/L, multiply by 0.331). Participants were randomized to 1 of 3 treatment groups (metformin, metformin plus rosiglitazone, or metformin with lifestyle intervention) and followed for 2 to 6 years (2004-2011) to assess treatment effects on primary (time to treatment failure) and secondary (complications) outcomes.^12^ After the TODAY trial, 572 individuals were enrolled in the TODAY2 observational follow-up study (2014-2020); they received care in their communities and underwent annual assessments. Publications from TODAY and TODAY2 are available online.^19^

The TODAY2 *i*Count ancillary study (July 2017 to February 2019) enrolled participants aged 19 to 31 years, with independent data collection and access to TODAY2 assessment data. Participants were recruited by staff at an annual TODAY2 visit and were informed about *i*Count procedures, detailed in a signed consent document. All other assessments (eg, HbA_1c_ levels and complications or comorbidities) were performed per TODAY2 protocols.^12^ Participants completed psychosocial questionnaires at the *i*Count enrollment visit (baseline [T1]) and at the subsequent 1-year TODAY2 annual visit (follow-up [T2]). Participants received financial compensation for assessment time.

### Measures

Data on participant characteristics were obtained using a self-report questionnaire and included the following: age, gender, race and ethnicity, years of education, annual income, employment status, duration of diabetes, and diabetes in the nuclear family. Race and ethnicity were included because it is well established that average glycemic control varies across racial and ethnic groups. These data are reported as Hispanic, non-Hispanic Black (hereinafter, Black), non-Hispanic White (hereinafter, White), or other race or ethnicity (American Indian or Alaska Native or Asian). Participant BMI, HbA_1c_ (analyzed at the University of Washington Northwest Lipid Research Laboratories in Seattle), and number of comorbidities or complications (ie, hypertension, neuropathy, dyslipidemia, kidney disease, or retinopathy) were assessed per TODAY2 standardized protocol procedures.^12^ Outcomes of interest were high HbA_1c_ (≥8.0%) at T1 and T2, continuous HbA_1c_ level at T1 and T2, and change in HbA_1c_ from T1 to T2 (decrease ≥0.5%, −0.5% to 0.5%, or increase ≥0.5%) (to convert to proportion of total hemoglobin, multiply by 0.01).

### Psychosocial Measures

Eight psychosocial measures were used in this study. Psychological factors were assessed with the Diabetes Self-Efficacy Scale (DSES), the Beliefs About Medicines Questionnaire (BMQ), the Problem Areas in Diabetes Scale–5 (PAID-5), the Diabetes Attitude Scale (DAS), the Patient Health Questionnaire–8 (PHQ-8), and the Generalized Anxiety Disorder Questionnaire–7 (GAD-7). Social factors were assessed with the Chronic Illness Resources Survey (CIRS) and the Material Needs Insecurities Survey (MNIS).

The 8-item DSES (range, 8-80)^20^ measures how confident one is in their ability to perform self-care behaviors. Higher scores reflect greater diabetes self-efficacy. The DSES scores were rescaled to align with other measures ranging from 1 to 5 by dividing by 5. Each point on this rescaled measure represents a 5-point change on the original continuous scale.

On the BMQ (range, 5-25),^21^ two 5-item scales measure beliefs in the necessity of diabetes medicines (eg, “My medicines protect me from getting worse”) and concerns about diabetes medicines (eg, “I worry about the long-term effects of my medicines”). Two 4-item scales (range, 4-20) measure beliefs that medicines are harmful (eg, “Medicines do more harm than good”) or overused (eg, “Doctors use too many medicines”). Higher scores reflect stronger beliefs. Subscale scores were rescaled to align with other measures ranging from 1 to 5 by dividing by 5. Each point on this rescaled measure represents a 5-point change on the original continuous scale.

The 5-item PAID-5 (range, 0-20)^22^ measures diabetes-related emotional distress using a list of common diabetes-related concerns. Respondents indicate the degree to which each concern is a problem, and higher scores reflect greater diabetes distress (continuous). A score of 8 or greater defines distress warranting further assessment (ie, high diabetes distress; categorical).

The 33-item DAS^23^ is a continuous scale that measures attitudes toward diabetes. We used 3 DAS subscales (range, 1-5): perceived seriousness of diabetes, its psychosocial impact, and attitudes toward patient autonomy. Higher numbers indicate greater perceived seriousness and impact and a positive attitude toward patient autonomy (ie, the right to decide how hard one works to control blood glucose).

The 8-item PHQ-8 (range, 0-20)^24^ measures the presence and severity of depressive symptoms in the prior 2 weeks. Higher scores reflect a greater number of and greater severity of depressive symptoms (continuous). A PHQ-8 score of 10 or greater is a screen for major depressive disorder, which we refer to as moderate to severe depressive symptoms here (categorical).

The 7-item GAD-7 (range, 0-21)^25^ measures the presence and severity of anxiety disorder symptoms over the prior 2 weeks. Higher scores reflect a greater number of and greater severity of anxiety symptoms (continuous). A GAD-7 score of 10 or greater is a screen for anxiety disorders, which we refer to as moderate to severe anxiety symptoms here (categorical).

The 22-item CIRS^26,27^ is a continuous scale (range, 1-5) that measures multilevel self-management support, including support from one’s family or friends, neighborhood, and larger community. The CIRS is a list of available resources that support self-care, and respondents indicate how often they have used each resource over the prior 6 months. Higher scores indicate more support.

The MNIS^28^ is composed of validated measures of several material need insecurities or social determinants of health. We used subscales assessing food, housing, and medication insecurities (eAppendix in Supplement 1). Participants also completed a health care usage survey that asked, “In the past 12 months, were you covered by a health care plan?” Lack of health care coverage was a fourth insecurity. Respondents indicated whether each need was met due to cost (categorical) over the previous 12 months.

### Statistical Analysis

Demographics, clinical parameters, and psychosocial measures are summarized as means (SDs) for continuous variables or as proportions for categorical variables. Multivariable logistic regression models evaluated the independent association of each psychosocial factor with the likelihood of an HbA_1c_ level of 8.0% or greater at T1 and T2. We chose 8.0% as the cutoff defining high HbA_1c_ because this was the benchmark for loss of glycemic control in the TODAY study and is clinically meaningful. Multivariable linear regression models assessed associations with HbA_1c_ level at T1 and T2, a continuous variable. Separate models were fit for each psychosocial factor. All models were adjusted for race and ethnicity, employment status, BMI, number of comorbidities and complications, and diabetes in the nuclear family. Additional covariates were identified from univariate analyses and therefore varied based on the specific analysis. In cross-sectional analyses of T1 HbA_1c_ groups, age was also a covariate. Analyses of T1 HbA_1c_ level included age, education, and income as adjustments. In the longitudinal analyses of T2 HbA_1c_ groups and HbA_1c_ level, education was included. We also performed analyses comparing 3 groups: those whose HbA_1c_ decreased 0.5% or more, those whose HbA_1c_ increased 0.5% or more, and those whose HbA_1c_ remained stable (ie, change between −0.5% and 0.5%; eTable 3 in Supplement 1). In these adjusted analyses, BMI and number of complications were added. Finally, we explored whether changes in psychosocial factors over time were associated with T2 HbA_1c_ levels. *P* < .05 (2-sided) was considered statistically significant. All analyses and *P* values were considered exploratory per TODAY and *i*Count Study Group analysis plans. Analyses were performed using SAS, version 9.4 (SAS Institute Inc). Data were analyzed from December 2021 to September 2023.

## Results

### Participants

Of the 411 eligible TODAY2 participants approached, 381 consented to participate in the *i*Count study. Of the 381 enrolled, 348 had HbA_1c_ measurements at both T1 and T2 and comprised the analysis cohort (eFigure in Supplement 1). The analysis group included 229 women (65.8%) and 119 men (34.2%). Their mean (SD) age was 26.1 (2.5) years, and most (269 [77.3%]) were employed or students. A total of 131 participants (37.6%) were Black, 127 (36.5%) were Hispanic, 71 (20.4%) were White, and 19 (5.5%) were of other race or ethnicity. Most *i*Count participants were from economically disadvantaged populations. A total of 209 (74.6%) reported at least 1 unmet need, 267 (76.7%) had a high school diploma or fewer years of education, and 265 (82.0%) had an annual income less than $35 000 (Table 1). On average, participants had poor glycemic control (mean [SD] HbA_1c_, 9.4% [2.8%]), with a mean (SD) of 2.5 (1.4) complications. We compared the analysis cohort with the original TODAY cohort on selected demographic variables and found no statistically significant differences (eTable 1 in Supplement 1). We also compared the analysis cohort with participants who were recruited to the *i*Count study but excluded due to missing T2 HbA_1c_ levels. Excluded individuals had a slightly shorter diabetes duration (mean [SD], 11.8 [1.6] vs 12.5 [1.5] years; *P* = .04) and were more likely to be of other race or ethnicity (7 [21.2%] vs 19 [5.5%]; overall *P* = .007) (eTable 2 in Supplement 1).

**Table 1.** Participant Characteristics at T1 (Baseline)^a^

Characteristic	All participants (N = 348)	High HbA_1c_ (≥8.0%) group (n = 227)	Low HbA_1c_ (<8.0%) group (n = 121)	*P* value^b^
**Demographic**				
Age, mean (SD), y	26.1 (2.5)	25.9 (2.5)	26.5 (2.4)	.03
Gender				
Female	229 (65.8)	148 (65.2)	81 (66.9)	.81
Male	119 (34.2)	79 (34.8)	440 (33.1)	
Race and ethnicity				
Hispanic	127 (36.5)	94 (41.4)	33 (27.3)	.003
Non-Hispanic Black	131 (37.6)	88 (38.8)	43 (35.5)	
Non-Hispanic White	71 (20.4)	35 (15.4)	36 (29.8)	
Other^c^	19 (5.5)	10 (4.4)	9 (7.4)	
Education				
Less than high school diploma	32 (9.2)	24 (10.6)	8 (6.6)	.08
High school or trade school diploma	235 (67.5)	158 (69.6)	77 (63.6)	
Associate’s degree or greater	81 (23.3)	45 (19.8)	36 (29.8)	
Annual income, $				
<34 999	265 (82.0)	177 (84.7)	88 (77.2)	.10
≥35 000	58 (18.0)	32 (15.3)	26 (22.8)	
Employment status				
Employed or student	269 (77.3)	166 (73.1)	103 (85.1)	.01
Unemployed or disabled	79 (22.7)	61 (26.9)	18 (14.9)	
BMI, mean (SD)	36.3 (8.5)	35.5 (8.0)	37.9 (9.3)	.02
Diabetes duration, mean (SD), y	12.5 (1.5)	12.4 (1.5)	12.7 (1.5)	.08
HbA_1c_, mean (SD), %	9.4 (2.8)	11.1 (1.8)	6.2 (0.8)	<.001
Comorbidities or complications, mean (SD)	2.5 (1.4)	2.8 (1.3)	1.9 (1.4)	<.001
Diabetes in the nuclear family	203 (59.5)	145 (65.3)	58 (48.7)	.004
Health care coverage				
No	48 (13.8)	33 (14.5)	15 (12.4)	.63
Yes	300 (86.2)	194 (85.5)	106 (87.6)	
**Psychological factor**				
Diabetes attitudes, mean (SD)^d^				
Seriousness of diabetes	4.0 (0.5)	4.0 (0.5)	4.0 (0.6)	.75
Psychosocial impact	4.0 (0.6)	4.0 (0.6)	4.0 (0.6)	.31
Patient autonomy	3.7 (0.5)	3.7 (0.5)	3.8 (0.5)	.31
Beliefs about medicines, mean (SD)^e^				
Specific beliefs				
Necessity	13.2 (9.1)	14.2 (8.9)	11.4 (9.4)	.01
Concerns	9.8 (7.2)	10.6 (7.2)	8.2 (7.0)	.002
General beliefs				
Harm	9.6 (3.0)	9.7 (3.0)	9.5 (3.1)	.62
Overuse	10.1 (3.1)	10.1 (3.0)	10.1 (3.3)	.88
Diabetes self-efficacy, mean (SD)^f^	54.1 (16.2)	53.1 (15.9)	55.9 (16.6)	.12
Diabetes distress^g^				
Score, mean (SD)	4.8 (4.8)	5.6 (4.8)	3.4 (4.3)	<.001
Participants with high diabetes distress	86 (24.7)	70 (30.8)	16 (13.2)	<.001
Depression symptoms^h^				
Score, mean (SD)	3.2 (4.2)	3.0 (4.2)	3.5 (4.2)	.25
Severity				
None to mild	309 (90.4)	204 (91.9)	105 (87.5)	.25
Moderate to severe	33 (9.6)	18 (8.1)	15 (12.5)	
Anxiety symptoms^i^				
Score, mean (SD)	2.4 (3.8)	2.4 (3.9)	3.5 (4.2)	.95
Severity				
None to mild	322 (94.2)	209 (94.1)	113 (94.2)	>.99
Moderate to severe	20 (5.8)	13 (5.9)	7 (5.8)	
**Social factor**				
Self-management support, mean (SD)^j^	2.6 (0.7)	2.5 (0.7)	2.7 (0.7)	.11
Material need insecurity^k^				
Medication	65 (35.3)	44 (35.8)	21 (34.4)	>.99
Food	130 (44.2)	86 (45.5)	44 (41.9)	.62
Housing	96 (27.6)	49 (29.5)	29 (24.0)	.31
Health care coverage	48 (13.8)	33 (14.5)	15 (12.4)	.63
≥1 Insecurity^l^	209 (74.6)	141 (77.1)	68 (70.1)	.25
≥2 Insecurities^l^	90 (30.4)	62 (31.2)	28 (28.9)	.79

Abbreviations: BMI, body mass index (calculated as weight in kilograms divided by height in meters squared); HbA_1c_, hemoglobin A_1c_ (to convert to proportion of total hemoglobin, multiply by 0.01).

^a^ Unless indicated otherwise, values are presented as No. (%) of participants. Percentages are calculated based on the available data for each variable.

^b^ Groups were compared on these characteristic variables using the *t* test (continuous) and Fisher exact test or χ^2^ test (categorical).

^c^ Includes American Indian or Alaska Native or Asian.

^d^ Assessed using the Diabetes Attitude Scale (subscale range, 1-5; higher subscale scores indicate greater belief that diabetes is a serious disease, that diabetes has had a greater impact on quality of life, and that persons with diabetes have a right to decide how hard they will work to control their blood glucose).

^e^ Assessed using 2 scales from the Beliefs About Medicines Questionnaire that measure beliefs in the necessity of diabetes medicines and concerns about diabetes medicines (range, 5-25; a higher score on each subscale indicates greater belief that diabetes medicines are necessary and more concerns about them) and using 2 scales that measure beliefs that, in general, medicines are harmful or overused (range, 4-20; a higher score indicates greater belief that medicines are harmful or overused).

^f^ Assessed using the Diabetes Self-Efficacy Scale (range, 8-80; a higher score indicates greater diabetes self-efficacy).

^g^ Assessed using the Problem Areas in Diabetes Scale–5 (range, 0-20; a score ≥8 indicates high diabetes distress).

^h^ Assessed using the Patient Health Questionnaire–8 (range, 0-20; a score ≥10 indicates moderate to severe depressive symptoms).

^i^ Assessed using the Generalized Anxiety Disorder Questionnaire–7 (range, 0-21; a score ≥10 indicates moderate to severe anxiety symptoms).

^j^ Assessed using the Chronic Illness Resources Survey (range, 1-5; a higher score indicates greater use of support for diabetes self-management).

^k^ Assessed using the Material Needs Insecurities Survey. Data are presented for participants who reported the insecurity was present.

^l^ Included those who had missing medication insecurity but had reported 1 or more food, housing, or health care coverage insecurities.

### Participant Factors

#### High (≥8.0%) vs Low (<8.0%) HbA_1c_ Group Comparisons

Participants in the high HbA_1c_ group were significantly younger than those in the low HbA_1c_ group (mean [SD], 25.9 [2.5] vs 26.5 [2.4] years; *P* = .03) and had higher rates of unemployment or disability (61 [77.2%] vs. 18 [22.8%]; *P* = .008). In addition, participants in the high HbA_1c_ group were more likely to be racial or ethnic minority individuals: 67.2% of Black participants (n = 88), 74.0% of Hispanic participants (n = 94), and 52.6% of participants (n = 10) of other race or ethnicity had high HbA_1c_ compared with 49.3% of White participants (n = 35) (*P* = .003). The high HbA_1c_ group had a lower mean (SD) BMI (35.5 [8.0] vs 37.9 [9.3]; *P* = .02), a greater mean (SD) number of complications (2.8 [1.3] vs 1.9 [1.4]; *P* < .001), and a higher percentage of participants with a family member with diabetes (145 [71.4%] vs 58 [28.6%]; *P* = .004) (Table 1).

#### Change in HbA_1c_ Group Comparisons (Decrease ≥0.5% vs Stable vs Increase ≥0.5%)

Group mean (SD) HbA_1c_ did not change significantly from T1 to T2 (9.3% [2.8%] vs 9.3% [2.7%]; *P* = .60). In the comparison of participants whose HbA_1c_ decreased 0.5% or more (108 [31.0%]) vs increased 0.5% or more (129 [37.1%]) or remained stable (111 [31.9%]), the stable group had the highest mean (SD) BMI (38.5 [9.6] vs 35.1 [7.4] vs 35.4 [8.2]; *P* = .008) and the lowest mean (SD) number of complications (2.2 [1.5] vs 2.7 [1.3] vs 2.6 [1.3]; *P* = .03) (eTable 3 in Supplement 1).

### Psychosocial Factors Associated With High HbA_1c_ (T1 and T2)

#### T1 Results

In adjusted logistic regression analyses of psychosocial factors associated with high HbA_1c_ (≥8.0%) at T1, beliefs about medicines, diabetes distress, and self-management support were associated with high T1 HbA_1c_ (Table 2). Specifically, every 5-point higher BMQ-necessity beliefs and concerns score increased the odds of high HbA_1c_ 1.2 and 1.3 times (adjusted odds ratio [OR], 1.23 [95% CI, 1.07-1.43]; *P* = .004; OR, 1.33 [95% CI, 1.10-1.61]; *P* = .003). Every 1-point higher PAID-5 diabetes distress score and high diabetes distress increased the odds of high HbA_1c_ 1.1 and 2.7 times (OR, 1.10 [95% CI, 1.04-1.17]; *P* = .002; OR, 2.72 [95% CI, 1.37-5.38]; *P* = .004). Finally, every 1-point higher CIRS self-management support score decreased the odds of high HbA_1c_ by 40.0% (OR, 0.60 [95% CI, 0.41-0.88]; *P* = .009).

**Table 2.** Psychosocial Factors Associated With HbA_1c_ Groups (High vs Low) at T1 (Baseline)

Psychosocial measure	OR (95% CI)^a^	*P* value
**Psychological factor**		
Diabetes attitudes, per 1-point increase^b^		
Seriousness of diabetes	1.07 (0.66-1.75)	.77
Psychosocial impact	1.25 (0.79-1.97)	.34
Patient autonomy	0.82 (0.49-1.37)	.45
Beliefs about medicines, per 5-point increase^c^		
Specific beliefs		
Necessity	1.23 (1.07-1.43)	.004
Concerns	1.33 (1.10-1.61)	.003
General beliefs		
Harm	1.35 (0.88-2.09)	.17
Overuse	1.10 (0.73-1.66)	.65
Diabetes self-efficacy, per 5-point increase^d^	0.95 (0.87-1.02)	.16
Diabetes distress^e^		
Per 1-point increase	1.10 (1.04-1.17)	.002
Score ≥8 (vs <8)	2.72 (1.37-5.38)	.004
Depression symptoms^f^		
Moderate to severe (vs none to mild symptoms)	0.42 (0.17-1.01)	.05
Anxiety symptoms^g^		
Moderate to severe (vs none to mild symptoms)	0.90 (0.29-2.79)	.86
**Social factor**		
Self-management support, per 1-point increase^h^	0.60 (0.41-0.88)	.009
Material need insecurity^i^		
Medication	1.19 (0.58-2.45)	.64
Food	1.34 (0.76-2.37)	.31
Housing	1.19 (0.67-2.13)	.55
Health care coverage	0.78 (0.36-1.71)	.54
≥1 Insecurity	1.46 (0.78-2.73)	.24
≥2 Insecurities	1.13 (0.62-2.04)	.69

Abbreviations: BMI, body mass index (calculated as weight in kilograms divided by height in meters squared); HbA_1c_, hemoglobin A_1c_ (to convert to proportion of total hemoglobin, multiply by 0.01); OR, odds ratio.

^a^ Of the 348 participants, 227 (65.2%) had high HbA_1c_ (≥8.0%) and 121 (34.8%) had low HbA_1c_ (<8.0%) at T1. All models were adjusted for age, race and ethnicity, employment status, BMI, number of comorbidities or complications, and diabetes in the nuclear family at T1.

^b^ Assessed using the Diabetes Attitude Scale (subscale range, 1-5; higher subscale scores indicate greater belief that diabetes is a serious disease, that diabetes has had a greater impact on quality of life, and that persons with diabetes have a right to decide how hard they will work to control their blood glucose).

^c^ Assessed using 2 scales from the Beliefs About Medicines Questionnaire that measure beliefs in the necessity of diabetes medicines and concerns about diabetes medicines (range, 5-25; higher scores indicate greater belief that diabetes medicines are necessary and more concerns about them) and 2 scales that measure beliefs that, in general, medicines are harmful or overused (range, 4-20; a higher score indicates greater belief that medicines are harmful or overused).

^d^ Assessed using the Diabetes Self-Efficacy Scale (range, 8-80; a higher score indicates greater diabetes self-efficacy).

^e^ Assessed using the Problem Areas in Diabetes Scale–5 (range, 0-20; a score ≥8 indicates high diabetes distress).

^f^ Assessed using the Patient Health Questionnaire–8 (range, 0-20; a score ≥10 indicates moderate to severe depressive symptoms).

^g^ Assessed using the Generalized Anxiety Disorder Questionnaire–7 (range, 0-21; a score ≥10 indicates moderate to severe anxiety symptoms).

^h^ Assessed using the Chronic Illness Resources Survey (range, 1-5; a higher score indicates greater use of support for diabetes self-management).

^i^ Assessed using the Material Needs Insecurities Survey. Comparisons are among participants reporting vs not reporting the insecurity.

#### T2 Results

Adjusted analyses of psychosocial factors associated with high T2 HbA_1c_ yielded similar findings (Table 3). Every 5-point higher BMQ-necessity beliefs and concerns score increased the odds of high T2 HbA_1c_ 1.2 times (OR, 1.19 [95% CI, 1.03-1.37]; *P* = .02; OR, 1.20 [95% CI, 1.00-1.45]; *P* = .049). Every 1-point higher diabetes distress score and high diabetes distress increased the odds of high T2 HbA_1c_ (OR, 1.08 [95% CI, 1.02-1.15]; *P* = .006; OR, 2.18 [95% CI, 1.15-4.13]; *P* = .02). Every 1-point higher CIRS self-management support score decreased the odds of high T2 HbA_1c_ by 30.0% (OR, 0.67 [95% CI, 0.46-0.97]; *P* = .04). Additionally, diabetes self-efficacy emerged as a statistically significant factor. Every 5-point higher DSES score decreased the odds of high T2 HbA_1c_ by 10.0% (OR, 0.91 [95% CI, 0.84-0.99]; *P* = .02).

**Table 3.** Psychosocial Factors Associated With HbA_1c_ Groups at T2 (1-Year Follow-Up)

Psychosocial measure	OR (95% CI)^a^	*P* value
**Psychological factor**		
Diabetes attitudes, per 1-point increase^b^		
Seriousness of diabetes	0.80 (0.49-1.31)	.37
Psychosocial impact	1.05 (0.66-1.65)	.84
Patient autonomy	1.52 (0.90-2.57)	.12
Beliefs about medicines, per 5-point increase^c^		
Specific beliefs		
Necessity	1.19 (1.03-1.37)	.02
Concerns	1.20 (1.00-1.45)	.049
General beliefs		
Harm	1.32 (0.86-2.03)	.20
Overuse	1.22 (0.81-1.83)	.34
Diabetes self-efficacy, per 5-point increase^d^	0.91 (0.84-0.99)	.02
Diabetes distress^e^		
Per 1-point increase	1.08 (1.02-1.15)	.006
Score ≥8 (vs <8)	2.18 (1.15-4.13)	.02
Depression symptoms^f^		
Moderate to severe (vs none to mild symptoms)	1.16 (0.48-2.82)	.74
Anxiety symptoms^g^		
Moderate to severe (vs none to mild symptoms)	0.77 (0.26-2.27)	.63
**Social factor**		
Self-management support, per 1-point increase^h^	0.67 (0.46-0.97)	.04
Material need insecurity^i^		
Medication	1.46 (0.73-2.93)	.29
Food	1.32 (0.75-2.31)	.34
Housing	1.31 (0.73-2.33)	.37
Health care coverage	0.74 (0.34-1.61)	.45
≥1 Insecurity	0.92 (0.49-1.74)	.80
≥2 Insecurities	1.46 (0.81-2.64)	.21

Abbreviations: BMI, body mass index (calculated as weight in kilograms divided by height in meters squared); HbA_1c_, hemoglobin A_1c_ (to convert to proportion of total hemoglobin, multiply by 0.01); OR, odds ratio.

^a^ Of the 348 participants, 220 (63.2%) had high HbA_1c_ (≥8.0%) and 128 (36.8%) had low HbA_1c_ (<8.0%) at T2. All logistic models were adjusted for race and ethnicity, education, employment status, BMI, number of comorbidities or complications, and diabetes in the nuclear family at T1 (baseline).

^b^ Assessed using the Diabetes Attitude Scale (subscale range, 1-5; higher subscale scores indicate greater belief that diabetes is a serious disease, that diabetes has had a greater impact on quality of life, and that persons with diabetes have a right to decide how hard they will work to control their blood glucose).

^c^ Assessed using 2 scales from the Beliefs About Medicines Questionnaire that measure beliefs in the necessity of diabetes medicines and concerns about diabetes medicines (range, 5-25; higher scores indicate greater belief that diabetes medicines are necessary and more concerns about them) and using 2 scales that measure beliefs that, in general, medicines are harmful or overused (range, 4-20; higher scores indicate greater belief that medicines are harmful or overused).

^d^ Assessed using the Diabetes Self-Efficacy Scale (range, 8-80; a higher score indicates greater diabetes self-efficacy).

^e^ Assessed using the Problem Areas in Diabetes Scale–5 (range, 0-20; a score ≥8 indicates high diabetes distress).

^f^ Assessed using the Patient Health Questionnaire–8 (range, 0-20; a score ≥10 indicates moderate to severe depressive symptoms).

^g^ Assessed using the Generalized Anxiety Disorder Questionnaire–7 (range, 0-21; a score ≥10 indicates moderate to severe anxiety symptoms).

^h^ Assessed using the Chronic Illness Resources Survey (range, 1-5; a higher score indicates greater use of support for diabetes self-management).

^i^ Assessed using the Material Needs Insecurities Survey. Comparisons are among participants reporting vs not reporting the insecurity.

### Psychosocial Factors Associated With HbA_1c_ Level (T1 and T2)

#### T1 Results

In adjusted linear regression analyses of psychosocial factors associated with T1 HbA_1c_ level, diabetes distress was a significant factor. For every 1-point higher diabetes distress score, T1 HbA_1c_ was higher by 0.09 (*P* = .01) (eTable 4 in Supplement 1).

#### T2 Results

In adjusted analyses of psychosocial factors associated with T2 HbA_1c_, diabetes distress was again significant. For every 1-point higher T1 diabetes distress score, T2 HbA_1c_ was higher by 0.08 (95% CI, 0.02-0.13; *P* = .01) (Table 4).

**Table 4.** Multivariable Analyses of Psychosocial Factors Associated With HbA_1c_ Level at T2 (1-Year Follow-Up)

Psychosocial measure	Estimate (95% CI)^a^	SE^a^	*P* value
**Psychological factor**			
Diabetes attitudes, per 1-point increase^b^			
Seriousness of diabetes	−0.02 (−0.55 to 0.51)	0.27	.95
Psychosocial impact	0.16 (−0.33 to 0.64)	0.25	.52
Patient autonomy	0.39 (−0.15 to 0.93)	0.27	.16
Beliefs about medicines, per 5-point increase^c^			
Specific beliefs			
Necessity	0.08 (−0.07 to 0.23)	0.08	.30
Concerns	0.09 (−0.10 to 0.28)	0.10	.35
General beliefs			
Harm	0.39 (−0.06 to 0.84)	0.23	.09
Overuse	0.28 (−0.15 to 0.71)	0.22	.20
Diabetes self-efficacy, per 5-point increase^d^	−0.04 (−0.13 to 0.04)	0.04	.31
Diabetes distress^e^			
Per 1-point increase	0.08 (0.02 to 0.13)	0.03	.01
Score ≥8 (vs <8)	0.59 (−0.04 to 1.23)	0.32	.07
Depression symptoms^f^			
Moderate to severe (vs none to mild symptoms)	−0.09 (−1.03 to 0.84)	0.48	.84
Anxiety symptoms^g^			
Moderate to severe (vs none to mild symptoms)	0.14 (−0.97 to 1.25)	0.56	.80
**Social factor**			
Self-management support, per 1-point increase^h^	−0.31 (−0.69 to 0.07)	0.19	.11
Material need insecurity^i^			
Medication	0.45 (−0.33 to 1.23)	0.40	.26
Food	0.25 (−0.35 to 0.84)	0.30	.42
Housing	0.55 (−0.05 to 1.16)	0.31	.07
Health care coverage	−0.12 (−0.93 to 0.69)	0.41	.78
≥1 Insecurity	0.36 (−0.33 to 1.05)	0.35	.30
≥2 Insecurities	0.41 (−0.24 to 1.06)	0.33	.22

Abbreviations: BMI, body mass index (calculated as weight in kilograms divided by height in meters squared); HbA_1c_, hemoglobin A_1c_ (to convert to proportion of total hemoglobin, multiply by 0.01).

^a^ Of the 348 participants, 220 (63.2%) had high HbA_1c_ (≥8.0%) and 128 (36.8%) had low HbA_1c_ (<8.0%) at T2. All generalized linear models were adjusted for age, race and ethnicity, education, income, employment status, BMI, number of comorbidities or complications, and diabetes in the nuclear family at T1.

^b^ Assessed using the Diabetes Attitude Scale (subscale range, 1-5; higher scores indicate greater belief that diabetes is a serious disease, that diabetes has had a greater impact on quality of life, and that persons with diabetes have a right to decide how hard they will work to control their blood glucose).

^c^ Assessed using 2 scales from the Beliefs About Medicines Questionnaire that measure beliefs in the necessity of diabetes medicines and concerns about diabetes medicines (range, 5-25; higher scores indicate greater belief that diabetes medicines are necessary and more concerns about them) and using 2 scales that measure beliefs that, in general, medicines are harmful or are overused (range, 4-20; higher scores indicate greater belief that medicines are harmful or overused).

^d^ Assessed using the Diabetes Self-Efficacy Scale (range, 8-80; a higher score indicates greater diabetes self-efficacy).

^e^ Assessed using the Problem Areas in Diabetes Scale–5 (range, 0-20; a score ≥8 indicates high diabetes distress).

^f^ Assessed using the Patient Health Questionnaire–8 (range, 0-20; a score ≥10 indicates moderate to severe depressive symptoms).

^g^ Assessed using the Generalized Anxiety Disorder Questionnaire–7 (range, 0-21; a score ≥10 indicates moderate to severe anxiety symptoms).

^h^ Assessed using the Chronic Illness Resources Survey (range, 1-5; a higher score indicates greater use of support for diabetes self-management).

^i^ Assessed using the Material Needs Insecurities Survey. Comparisons are among participants reporting vs not reporting the insecurity.

### Psychosocial Factors Associated With Change in HbA_1c_

#### HbA_1c_ Change Group Results

In adjusted logistic regressions, BMQ-necessity beliefs and concerns scores were associated with higher odds of decreased T2 HbA_1c_ vs stable HbA_1c_ levels (Table 5). Specifically, every 5-point higher necessity beliefs and concerns score increased the odds of decreased HbA_1c_ 1.2 times (OR, 1.20 [95% CI, 1.03-1.39]; *P* = .02; OR, 1.22 [95% CI, 1.00- 1.47]; *P* = .048).

**Table 5.** Psychosocial Factors Associated With HbA_1c_ Change Groups Over Time

Psychosocial measure	HbA_1c_ decrease vs no change		HbA_1c_ increase vs no change		
	OR (95% CI)^a^	*P* value	OR (95% CI)^a^	*P* value
**Psychological factor**				
Diabetes attitudes, per 1-point increase^b^				
Seriousness of diabetes	1.01 (0.60- 1.70)	.97	0.87 (0.53-1.44)	.60
Psychosocial impact	0.99 (0.60-1.61)	.96	0.79 (0.50-1.27)	.33
Patient autonomy	1.28 (0.74-2.21)	.37	1.39 (0.82-2.35)	.22
Beliefs about medicines, per 5-point increase^c^				
Specific beliefs				
Necessity	1.20 (1.03-1.39)	.02	1.08 (0.94-1.25)	.28
Concerns	1.22 (1.00-1.47)	.048	1.10 (0.92-1.32)	.31
General beliefs				
Harm	1.17 (0.75-1.85)	.49	1.00 (0.65-1.56)	.98
Overuse	1.19 (0.76-1.86)	.44	1.19 (0.78-1.83)	.42
Diabetes self-efficacy, per 5-point increase^d^	0.98 (0.90-1.06)	.60	0.98 (0.90-1.06)	.54
Diabetes distress^e^				
Per 1-point increase	1.05 (0.99-1.11)	.11	1.03 (0.97-1.09)	.31
Score ≥8 (vs <8)	1.10 (0.58-2.09)	.77	1.07 (0.58-2.00)	.82
Depression symptoms^f^				
Moderate to severe (vs none to mild symptoms)	0.79 (0.29-2.11)	.63	1.17 (0.49-2.81)	.73
Anxiety symptoms^g^				
Moderate to severe (vs none to mild symptoms)	0.76 (0.24-2.38)	.64	0.56 (0.17-1.81)	.33
**Social factor**				
Self-management support, per 1-point increase^h^	0.93 (0.63-1.37)	.72	0.79 (0.54-1.16)	.23
Material need insecurity^i^				
Medication	0.99 (0.45-2.17)	.97	1.20 (0.56-2.58)	.65
Food	1.53 (0.84-2.80)	.17	1.16 (0.64-2.09)	.62
Housing	1.30 (0.69-2.44)	.42	1.70 (0.94-3.09)	.08
Health care coverage	0.61 (0.26-1.42)	.25	1.00 (0.47-2.12)	>.99
≥1 Insecurity	0.91 (0.45-1.83)	.79	0.94 (0.48-1.86)	.86
≥2 Insecurities	1.20 (0.62-2.32)	.58	1.42 (0.76-2.66)	.28

Abbreviations: BMI, body mass index (calculated as weight in kilograms divided by height in meters squared); HbA_1c_, hemoglobin A_1c_ (to convert to proportion of total hemoglobin, multiply by 0.01); OR, odds ratio.

^a^ Of the 348 participants, 108 (31.0%) had decreased HbA_1c_ by at least 0.5%, 129 (37.1%) had increased HbA_1c_ by at least 0.5%, and 111 (31.9%) had relatively stable HbA_1c_ over time. All multinomial logistic models were adjusted for baseline BMI and number of comorbidities or complications.

^b^ Assessed using the Diabetes Attitude Scale (subscale range, 1-5; higher subscale scores indicate greater belief that diabetes is a serious disease, that diabetes has had a greater impact on quality of life, and that persons with diabetes have a right to decide how hard they will work to control their blood glucose).

^c^ Assessed using 2 scales from the Beliefs About Medicines Questionnaire that measure beliefs in the necessity of diabetes medicines and concerns about diabetes medicines (range, 5-25; higher scores indicate greater belief that diabetes medicines are necessary and more concerns about them) and using 2 scales that measure beliefs that, in general, medicines are harmful or overused (range, 4-20; higher scores indicate greater belief that medicines are harmful or overused).

^d^ Assessed using the Diabetes Self-Efficacy Scale (range, 8-80; a higher score indicates greater diabetes self-efficacy).

^e^ Assessed using the Problem Areas in Diabetes Scale–5 (range, 0-20; a score ≥8 indicates high diabetes distress).

^f^ Assessed using the Patient Health Questionnaire–8 (range, 0-20; a score ≥10 indicates moderate to severe depressive symptoms).

^g^ Assessed using the Generalized Anxiety Disorder Questionnaire–7 (range, 0-21; a score ≥10 indicates moderate to severe anxiety symptoms).

^h^ Assessed using the Chronic Illness Resources Survey (range, 1-5; a higher score indicates greater use of support for diabetes self-management).

^i^ Assessed using the Material Needs Insecurities Survey. Comparisons are among participants reporting vs not reporting the insecurity.

#### HbA_1c_ Change as a Continuous Variable (Percent)

In adjusted analyses, none of the psychosocial factors were associated with change in HbA_1c_ level from T1 to T2. Additional details are provided in eTable 5 in Supplement 1.

#### Change in Psychosocial Factors and T2 HbA_1c_ Results

From T1 to T2, despite no planned intervention, mean (SD) scores on the DAS seriousness of diabetes subscale increased from T1 to T2 (4.03 [0.53] vs 4.10 [0.56]; *P* = .007), mean (SD) scores on the BMQ concerns subscale decreased (9.78 [7.18] vs 8.59 [7.08]; *P* = .004), the percentage of participants with moderate to severe anxiety symptoms increased (20 [5.7%] vs 33 [9.6%]; *P* = .02), and the percentage of participants with food insecurity decreased (130 [44.2%] vs 98 [34.3%]; *P* = .01). Other psychosocial factors were stable. Changes in psychosocial factors were not associated with T2 HbA_1c_, either categorically or continuously.

## Discussion

In this first (to our knowledge) longitudinal study of the association of psychosocial factors with glycemic control in young adults with youth-onset type 2 diabetes, beliefs about diabetes medicines were associated with HbA_1c_ over time. Individuals with greater belief that diabetes medicines are necessary and more concerns about diabetes medicines were significantly more likely to have high HbA_1c_ and to have at least a 0.5% decrease in HbA_1c_ (without planned intervention) 1 year later. Those with higher diabetes distress were more likely to have high HbA_1c_ and higher HbA_1c_ level 1 year later. Finally, those with higher diabetes self-efficacy or self-management support were less likely to have high HbA_1c_ 1 year later.

Beliefs about medicines have been studied within the Necessity-Concerns Framework to understand factors associated with medication adherence. This framework posits that adherence is affected by beliefs that medicines are necessary balanced against concerns about them.^29^ We previously reported that in a longitudinal study of young adults with youth-onset type 2 diabetes, having more concerns about diabetes medicines was associated with higher odds of low adherence to oral medications (ie, taking <80% of pills), greater belief that medicines are overused was associated with higher odds of low adherence to insulin, and harm and overuse beliefs were associated with a lower percentage of insulin adherence.^17^ However, we are unaware of any reports relating beliefs about medicines to HbA_1c_ in this group; therefore, we looked to research with older adults (adult-onset diabetes) for comparison. In one cross-sectional report of older adults with type 2 diabetes, necessity and concern beliefs about medicines were not associated with HbA_1c_, although they were associated with medication underuse.^30^ Noting the lack of publications that examine the relationships between these beliefs and HbA_1c_, our data provide compelling evidence of associations over time.

We found that lower diabetes self-efficacy was associated with high HbA_1c_ over time. Because of a lack of data relevant to this association from a comparable cohort, we looked to studies of those with adult-onset type 2 diabetes. A previously published meta-analysis of cross-sectional studies examined biobehavioral predictors of glycemic control in older adults.^6^ The investigators reported no association between self-efficacy and HbA_1c_, although self-efficacy was the most consistent predictor of adherence behaviors and was strongly related to dietary adherence, a consistent predictor of HbA_1c_.^6^ In contrast, our longitudinal data in young adults with youth-onset type 2 diabetes support an association between self-efficacy and HbA_1c_ levels over time.

Looking at diabetes distress among individuals with youth-onset type 2 diabetes, high diabetes distress was associated with higher HbA_1c_ levels in the TODAY2 cohort in cross-sectional analyses.^31^ There is a robust literature reporting cross-sectional associations between diabetes distress and HbA_1c_ among individuals with adult-onset type 2 diabetes,^32^ but there are few longitudinal studies. A prospective study (mean age, 57.8 years) reported cross-sectional associations between distress and HbA_1c_ but no associations in longitudinal analyses (9-month and 18-month follow-up).^33^ In contrast, our data support an association between distress and HbA_1c_ over time in this cohort. Our longitudinal finding that self-management support was associated with better glycemic control is also consistent with the extant literature on persons with adult-onset diabetes.^26^

We also assessed whether anxiety and depressive symptoms were associated with HbA_1c_ because cross-sectional studies of individuals with adult-onset type 2 diabetes often report associations between these factors and HbA_1c_, although the results are inconsistent.^2,34,35^ In the longitudinal study cited earlier,^33^ no association between depression and HbA_1c_ was found at 9-month and 18-month follow-up. Similarly, we found no associations.

Our finding that unmet material needs (ie, social determinants of health) were not associated with HbA_1c_ is in contrast with the adult-onset diabetes literature, in which unmet needs have been linked to negative diabetes-related outcomes.^36^ However, most *i*Count participants were from economically disadvantaged populations; as noted earlier, 209 (74.6%) reported at least 1 unmet need, 267 (76.7%) had a high school diploma or fewer years of education, and 265 (82.0%) had an annual income less than $35 000. The relatively low number of participants with no material need insecurities or financial stress likely limited our ability to find statistically significant differences based on unmet needs.

Young adults with type 2 diabetes experience many of the developmental challenges faced by peers with type 1 diabetes, such as changes in support networks, responsibilities, and health care systems.^37^ The few cross-sectional studies assessing the association of psychosocial factors with HbA_1c_ in young adults with type 1 diabetes reported that depression,^38^ diabetes distress,^39^ anxiety disorders,^40^ social support,^41^ and health care coverage insecurity^42^ were associated with HbA_1c_. However, because the management of type 1 diabetes frequently involves technologies (eg, insulin pumps and continuous glucose monitoring) that pose unique challenges, it is important to study the 2 groups separately and in longitudinal studies.

### Strengths and Limitations

Unlike most prior studies, these analyses were longitudinal and examined HbA_1c_ levels categorically (high vs low; decreased vs stable vs increased) and continuously (percentages). Notably, participants were members of the diverse and unique TODAY2 cohort, now young adults with youth-onset type 2 diabetes.

However, the study also has limitations. Participants recruited to the TODAY study may have differed from the general population with youth-onset type 2 diabetes in measured variables, including psychosocial factors. They had regular contact with TODAY staff over many years; individuals without that education and support might have higher HbA_1c_ levels. The small percentage of participants with symptoms of moderate to severe depression (9.6%) or anxiety (5.8%) and the high percentage with material needs may have limited power to find associations. Other unmeasured psychosocial factors (eg, health literacy) likely relate to HbA_1c_. Additional factors that can contribute to high HbA_1c_ (eg, health care practitioner–related clinical inertia^43^ or prescribing bias^44^) were not considered. Results may not generalize to countries with different health care systems.

The overarching aim of *i*Count was to identify targets for interventions to improve HbA_1c_. Beliefs about medicines may be targets, supporting beliefs that medicines are necessary to treat diabetes and addressing unvoiced concerns about them.

Diabetes self-efficacy is another potential target. There are many studies of behavior change programs that improve self-efficacy, including family-based,^45^ peer-supported,^46^ and lay-led^47^ interventions. However, self-efficacy is commonly a secondary, not primary, focus. Our data suggest that interventions that focus on enhancing self-efficacy as the primary outcome may be beneficial and may lead to other innovative approaches. Also, interventions could address high levels of diabetes distress and provide needed support for self-management. These data can inform future interventions; however, because this was not an intervention trial, we do not know whether addressing beliefs about medicines, ameliorating diabetes distress, enhancing self-efficacy, or promoting self-management support will lead to better glycemic control among individuals with youth-onset type 2 diabetes. Future research is needed to answer these questions.

## Conclusions

In this cohort study of young adults with youth-onset type 2 diabetes, psychosocial factors including beliefs about medicines, diabetes distress, diabetes self-efficacy, and self-management support were associated with HbA_1c_ levels over time. These data extend the Necessity-Concerns Framework of beliefs about medicines and medication adherence to glycemic control outcomes. Our results suggest that these potentially modifiable psychosocial factors may affect glycemic control, and thus the development of complications in this highly vulnerable group, but intervention trials are needed.

## Notes

Supplement 1. **eFigure.** *i*Count Participant Enrollment and Flow **eTable 1.** Baseline Characteristics of TODAY Randomized Clinical Trial and *i*Count Analysis Cohort **eTable 2.** Participant Characteristics for Those Included vs Excluded in Analyses **eTable 3.** Participant Characteristics of HbA_1c_ Change Groups (T1 to T2 Change) **eTable 4.** Psychosocial Factors Associated With HbA_1c_ Level at T1 **eTable 5.** Psychosocial Factors Associated With HbA_1c_ Level Change Over Time **eAppendix.** Material Needs Insecurities Survey

Supplement 2.
Data Sharing Statement
