# Supplementary material for: Psychosocial Factors and Glycemic Control in Young Adults With Youth-Onset Type 2 Diabetes
Source: JAMA Netw Open. 2024 Apr 8;7(4):e245620. doi: 10.1001/jamanetworkopen.2024.5620 (PMC11002701; doi:10.1001/jamanetworkopen.2024.5620)
Supplement: Supplement 1. — eFigure. iCount Participant Enrollment and Flow eTable 1. Baseline Characteristics of TODAY Randomized Clinical Trial and iCount Analysis Cohort eTable 2. Participant Characteristics for Those Included vs Excluded in Analyses eTable 3. Participant Characteristics of HbA1c Change Groups (T1 to T2 Change) eTable 4. Psychosocial Factors Associated With HbA1c Level at T1 eTable 5. Psychosocial Factors Associated With HbA1c Level Change Over Time eAppendix. Material Needs Insecurities Survey [file jamanetwopen-e245620-s001.pdf]

# Supplementary Online Content

Trief PM, Wen H, Burke B, et al. Psychosocial factors and glycemic control in young adults with youth-onset type 2 diabetes. *JAMA Netw Open*. 2024;7(4):e245620. doi:10.1001/jamanetworkopen.2024.5620

**eFigure.** *i*Count Participant Enrollment and Flow

**eTable 1.** Baseline Characteristics of TODAY Randomized Clinical Trial and *i*Count Analysis Cohort

**eTable 2.** Participant Characteristics for Those Included vs Excluded in Analyses

**eTable 3.** Participant Characteristics of $HbA_{1c}$ Change Groups (T1 to T2 Change)

**eTable 4.** Psychosocial Factors Associated With $HbA_{1c}$ Level at T1

**eTable 5.** Psychosocial Factors Associated With $HbA_{1c}$ Level Change Over Time

**eAppendix.** Material Needs Insecurities Survey

This supplementary material has been provided by the authors to give readers additional information about their work.

© 2024 Trief PM et al. *JAMA Network Open.*

**eFigure.** *i*Count Participant Enrollment and Flow

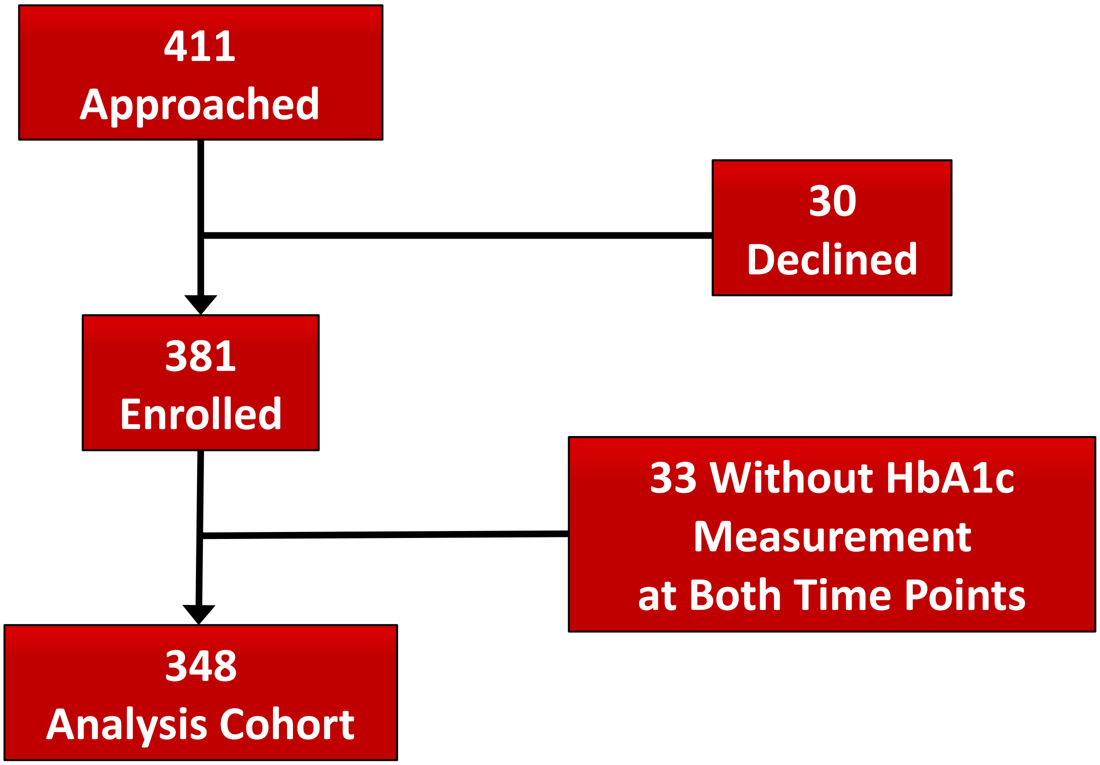


The iCount participants and all the participants originally enrolled in the TODAY randomized clinical trial did not differ at baseline by age, gender, race, ethnicity, body mass index, diabetes duration, or glycated hemoglobin A1c.

© 2024 Trief PM et al. *JAMA Network Open.*

**eTable 1.** Baseline Characteristics of TODAY Randomized Clinical Trial and *i*Count Analysis Cohort

| Characteristic | TODAY Clinical Trial Cohort (n = 699) | *i*Count Analysis Cohort (n = 348) | p-value [a] |
|---|---|---|---|
| Age, mean (SD), y | 14.0 (2.0) | 13.8 (2.0) | 0.95 |
| Gender, No. (%) | | | |
| Female | 452 (64.7) | 229 (65.8) | 0.73 |
| Male | 247 (35.3) | 119 (34.2) | |
| Race and Ethnicity, N (%) | | | |
| Black, non-Hispanic | 227 (32.5) | 131 (37.6) | 0.29 |
| Hispanic | 278 (39.8) | 127 (36.5) | |
| Other [b] | 52 (7.4) | 19 (5.5) | |
| White, non-Hispanic | 142 (20.3) | 71 (20.4) | |
| BMI, kg/m$^2$, mean(SD) | 34.9 (7.6) | 35.0 (7.7) | 0.93 |
| Diabetes duration, years, mean (SD) | 0.7 (0.5) | 0.7 (0.5) | 0.67 |
| HbA1c, %, mean (SD) | 6.0 (0.7) | 6.0 (0.8) | 0.76 |

Abbreviations: BMI, body mass Index (calculated as weight in kilograms divided by height in meters squared); HbA1c, glycated hemoglobin A1c.

[a] Groups were compared on these characteristic variables using t-test (continuous) and Fisher's Exact test (categorical).

[b] "Other" in Race/Ethnicity includes American Indian and non-Hispanic Asian individuals.

© 2024 Trief PM et al. *JAMA Network Open.*

**eTable 2.** Participant Characteristics for Those Included vs Excluded in Analyses

| | Total (n=381) | Included (n=348) | Excluded (n=33) | p-value[a] |
|---|---|---|---|---|
| Age, mean (SD), years | 26.0 (2.5) | 26.1 (2.5) | 25.2 (2.5) | 0.06 |
| Gender, No. (%) | | | | |
| Female | 259 (68.0) | 229 (65.8) | 30 (90.9) | 0.003 |
| Male | 122 (32.0) | 119 (34.2) | 3 (9.1) | |
| Race and Ethnicity, No. (%) | | | | |
| Black, non-Hispanic | 143 (37.5) | 131 (37.6) | 12 (36.4) | |
| Hispanic | 136 (35.7) | 127 (36.5) | 9 (27.3) | 0.007 |
| Other [b] | 26 (6.8) | 19 (5.5) | 7 (21.2) | |
| White, non-Hispanic | 76 (20.0) | 71 (20.4) | 5 (15.1) | |
| Education, No. (%) | | | | |
| <High school diploma | 38 (10.0) | 32 (9.2) | 6 (18.2) | 0.19 |
| High school or trade school | 257 (67.5) | 235 (67.5) | 22 (66.7) | |
| ≥Associate's degree or higher | 86 (22.5) | 81 (23.3) | 5 (15.1) | |
| Annual Income, $, No. (%) | | | | |
| <34,999 | 290 (82.4) | 265 (82.0) | 25 (86.2) | 0.57 |
| ≥35,000 | 62 (17.6) | 58 (18.0) | 4 (13.8) | |
| Employment status, No. (%) | | | | |
| Employed or student | 292 (76.6) | 269 (77.3) | 23 (36.4) | 0.32 |
| Unemployed/Disabled | 89 (23.4) | 79 (22.7) | 21 (63.6) | |
| BMI, kg/m$^2$ ,mean (SD) | 36.2 (8.4) | 36.3 (8.5) | 35.2 (6.4) | 0.43 |
| Diabetes duration, years, mean (SD) | 12.4 (1.5) | 12.5 (1.5) | 11.8 (1.6) | 0.04 |
| HbA1c, %, mean (SD) | 9.4 (2.8) | 9.4 (2.8) | 10.0 (2.4) | 0.24 |
| Comorbidities or complications, mean (SD) | 2.5 (1.4) | 2.5 (1.4) | 2.1 (1.2) | 0.10 |
| Diabetes in nuclear family, No. (%) | 224 (59.9) | 203 (59.5) | 21 (63.6) | 0.65 |
| Healthcare coverage, No. (%) | | | | |
| No | 55 (14.4) | 48 (13.8) | 7 (21.2) | 0.25 |
| Yes | 326 (85.6) | 300 (86.2) | 26 (79.8) | |

[a] Groups were compared on these characteristic variables using paired t-test (continuous) and Chi-Square test (categorical).
[b] Other" in Race/Ethnicity includes American Indian and non-Hispanic Asian individuals.

© 2024 Trief PM et al. *JAMA Network Open.*

**eTable 3.** Participant Characteristics of $HbA_{1c}$ Change Groups (T1 to T2 Change)

| Characteristic | Decrease ≥ 0.5% (n=108) | Between -0.5% and 0.5% (n=111) | Increase ≥ 0.5% (n=129) | p-value [a] |
|---|---|---|---|---|
| Age, mean (SD), years | 25.8 (2.2) | 26.4 (2.4) | 26.0 (2.7) | 0.10 |
| Gender, No. (%) | | | | |
| Female | 71 (65.7) | 71 (64.0) | 87 (67.4) | 0.85 |
| Male | 37 (34.3) | 40 (36.0) | 42 (32.6) | |
| Race and Ethnicity, No. (%) | | | | |
| Black, non-Hispanic | 42 (38.9) | 43 (38.7) | 46 (35.7) | 0.78 |
| Hispanic | 40 (37.0) | 36 (32.4) | 51 (39.5) | |
| Other [b] | 4 (3.7) | 9 (8.1) | 6 (4.6) | |
| White, non-Hispanic | 22 (20.4) | 23 (20.7) | 26 (20.2) | |
| Education, No. (%) | | | | |
| <High school diploma | 7 (6.5) | 10 (9.0) | 15 (11.6) | 0.63 |
| High school or trade school | 78 (72.2) | 73 (65.8) | 84 (65.1) | |
| ≥Associate's degree or higher | 23 (21.3) | 28 (25.2) | 30 (23.3) | |
| Annual Income, $, No. (%) | | | | |
| <34,999 | 84 (84.0) | 83 (79.8) | 98 (82.3) | 0.73 |
| ≥35,000 | 16 (16.0) | 21 (20.2) | 21 (17.7) | |
| Employment status, No. (%) | | | | |
| Employed or student | 82 (75.9) | 91 (82.0) | 96 (74.4) | 0.35 |
| Unemployed/Disabled | 26 (24.1) | 20 (18.0) | 33 (25.6) | |
| BMI, $kg/m^2$ ,mean (SD) | 35.1 (7.4) | 38.5 (9.6) | 35.4 (8.2) | 0.008 |
| Diabetes duration, years, mean (SD) | 12.4 (1.5) | 12.7 (1.5) | 12.3 (1.5) | 0.07 |
| HbA1c, %, mean (SD) | 11 (2.3) | 8.2 (2.9) | 9.0 (2.4) | <0.001 |
| Comorbidities or complications, mean (SD) | 2.7 (1.3) | 2.2 (1.5) | 2.6 (1.3) | 0.03 |
| Diabetes in nuclear family, No. (%) | 66 (62.3) | 58 (53.2) | 79 (62.7) | 0.26 |
| Healthcare coverage, No. (%) | | | | |
| No | 12 (11.1) | 15 (13.5) | 21 (16.3) | 0.51 |
| Yes | 96 (88.9) | 96 (86.5) | 108 (83.7) | |
| **Psychological Factors** | | | | |
| Diabetes attitudes[c], mean (SD) | | | | |
| Seriousness of diabetes | 4.1 (0.5) | 4.0 (0.5) | 4.0 (0.5) | 0.67 |
| Psychosocial impact | 4.0 (0.5) | 4.0 (0.6) | 3.9 (0.6) | 0.64 |
| Patient autonomy | 3.7 (0.5) | 3.7 (0.5) | 3.7 (0.6) | 0.56 |
| Beliefs about medicines[d], mean (SD) | | | | |
| Specific beliefs | | | | |
| Necessity | 14.9 (8.7) | 11.8 (9.3) | 13.1 (9.2) | 0.03 |
| Concerns | 10.9 (7.0) | 8.7 (7.3) | 9.8 (7.2) | 0.04 |
| General beliefs | | | | |
| Harm | 9.8 (3.2) | 9.6 (3.1) | 9.5 (2.9) | 0.78 |
| Overuse | 10.1 (3.2) | 9.9 (3.0) | 10.2 (3.1) | 0.82 |
| Diabetes self-efficacy[e], mean (SD) | 53.8 (14.9) | 54.9 (17.3) | 53.6 (16.3) | 0.71 |
| Diabetes distress[f] | | | | |
| Score, mean (SD) | 5.3 (4.8) | 4.1 (4.4) | 5.0 (5.1) | 0.12 |
| Participants with high diabetes distress, No. (%) | 28 (25.9) | 24 (21.6) | 34 (26.4) | 0.66 |

© 2024 Trief PM et al. *JAMA Network Open.*

**eTable 3.** Participant Characteristics of $HbA_{1c}$ Change Groups (T1 to T2) (continued)

| Characteristic | Decrease ≥ 0.5% (n=108) | Between -0.5% and 0.5% (n=111) | Increase ≥ 0.5% (n=129) | p-value [a] |
|---|---|---|---|---|
| Depression symptoms[g] | | | | |
| Score, mean (SD) | 2.6 (3.6) | 3.4 (4.6) | 3.5 (4.3) | 0.30 |
| Severity, No. (%) | | | | 0.64 |
| None-to-mild | 99 (92.5) | 97 (89.8) | 113 (89.0) | |
| Moderate-to-severe | 8 (7.5) | 11 (10.2) | 14 (11.0) | |
| Anxiety symptoms[h] | | | | |
| Score, mean (SD) | 2.2 (3.5) | 2.5 (4.1) | 2.6 (3.8) | 0.65 |
| Severity, No. (%) | | | | |
| None-to- mild | 101 (94.4) | 100 (92.6) | 121 (95.3) | 0.67 |
| Moderate-to-severe | 6 (5.6) | 8 (7.4) | 6 (4.7) | |
| **Social Factors** | | | | |
| Self-management support[i], mean (SD) | 2.6 (0.7) | 2.6 (0.7) | 2.5 (0.7) | 0.48 |
| Prevalence of material need insecurity[j], No. (%) | | | | |
| Medication | 21 (33.3) | 18 (34.6) | 26 (37.7) | 0.87 |
| Food | 45 (48.9) | 39 (40.6) | 46 (43.4) | 0.51 |
| Housing | 30 (27.8) | 24 (21.6) | 42 (32.6) | 0.17 |
| Healthcare coverage | 12 (11.1) | 15 (13.5) | 21 (16.3) | 0.54 |
| ≥1 insecurity[k] | 66 (73.3) | 64 (75.3) | 79 (75.2) | 0.94 |
| ≥2 insecurities[k] | 29 (30.9) | 23 (26.1) | 38 (33.3) | 0.54 |

[a] Groups were compared on these characteristic variables using t-test (continuous) and Fisher's Exact test or Chi-square (categorical).

[b] "Other" in Race/Ethnicity includes American Indian and non-Hispanic Asian individuals.

[c] Assessed using the Diabetes Attitudes Scale (subscale range, 1-5; higher scores indicate greater belief that diabetes is a serious disease, that diabetes has had a greater impact on quality of life, and that persons with diabetes have a right to decide how hard they will work to control their blood glucose, respectively).

[d] Assessed using the Beliefs About Medicines Questionnaire: 2 scales measure beliefs in the necessity of, and concerns about, diabetes medicines (range, 5-25; higher scores indicate more belief that diabetes medicines are necessary and more concerns about them, respectively) and 2 scales measure beliefs that, in general, medicines are harmful or overused (range, 4-20; higher score indicates more belief that medicines are harmful or overused).

[e] Assessed using the Diabetes Self-Efficacy Scale (range, 8-80; higher score indicates greater feelings of diabetes self-efficacy).

[f] Assessed using the Problem Areas in Diabetes Scale-5 (range, 0-20; a score ≥8 indicates high diabetes distress).

[g] Assessed using the Patient Health Questionnaire-8 (range, 0-20; score ≥10 indicates moderate-to-severe depressive symptoms).

[h] Assessed using the Generalized Anxiety Disorders Questionnaire-7 (range, 0-21; score ≥10 indicates moderate-to-severe anxiety symptoms).

[I] Assessed using the Chronic Illness Resources Survey (range, 1-5; higher score indicates greater use of support for diabetes self-management).

[j] Assessed using the Material Needs Insecurities Survey. Data are presented for participants who reported the insecurity was present.

[k] Included those who had missing medication insecurity but had reported one or more food, housing or healthcare coverage insecurities.

© 2024 Trief PM et al. *JAMA Network Open.*

**eTable 4.** Psychosocial Factors Associated With $HbA_{1c}$ Level at T1

| Psychosocial Measures | Estimates [a] | S.E. [a] | p-value |
|---|---|---|---|
| **Psychological Factors** | | | |
| Diabetes attitudes, per 1-point increase[b] | | | |
| Seriousness of diabetes | 0.09 | 0.29 | 0.74 |
| Psychosocial impact | 0.26 | 0.26 | 0.33 |
| Patient autonomy | 0.21 | 0.29 | 0.48 |
| Beliefs about medicines, per 5-point increase[c] | | | |
| Specific beliefs | | | |
| Necessity | 0.14 | 0.08 | 0.09 |
| Concerns | 0.14 | 0.10 | 0.17 |
| General beliefs | | | |
| Harm | 0.41 | 0.24 | 0.09 |
| Overuse | 0.15 | 0.23 | 0.51 |
| Diabetes self-efficacy, per 5-point increase [d] | -0.05 | 0.05 | 0.26 |
| Diabetes distress[e] | | | |
| Per 1-point increase | 0.09 | 0.03 | 0.01 |
| Score ≥8 (vs score <8) | 0.66 | 0.34 | 0.06 |
| Depression symptoms[f] | | | |
| Moderate-to-severe (vs None-to-mild symptoms) | -0.42 | 0.50 | 0.34 |
| Anxiety symptoms[g] | | | |
| Moderate-to-severe (vs None-to-mild symptoms) | 0.13 | 0.60 | 0.83 |
| **Social Factors** | | | |
| Self-management support, per 1-point increase[h] | -0.17 | 0.20 | 0.41 |
| Material need insecurity[i] | | | |
| Medication | 0.09 | 0.40 | 0.83 |
| Food | 0.31 | 0.31 | 0.33 |
| Housing | 0.48 | 0.33 | 0.15 |
| Healthcare coverage | -0.09 | 0.43 | 0.84 |
| ≥1 insecurity | 0.21 | 0.37 | 0.56 |
| ≥2 insecurities | 0.32 | 0.34 | 0.36 |

[a] Of 348 participants, 227 (65%) had high HbA1c (≥ 8%) and 121 (35%) had low HbA1c (< 8%) at T1. All generalized linear models were adjusted for age, race/ethnicity, education, annual income, employment status, BMI, no. of comorbidities/complications, and diabetes in nuclear family at T1.
[b] Assessed using the Diabetes Attitudes Scale (subscale range, 1-5; higher scores indicate greater belief that diabetes is a serious disease, that diabetes has had a greater impact on quality of life, and that persons with diabetes have a right to decide how hard they will work to control their blood glucose).
[c] Assessed using the Beliefs About Medicines Questionnaire: 2 scales measure beliefs in the necessity of, and concerns about, diabetes medicines (range, 5-25; higher scores indicate more belief that diabetes medicines are necessary and more concerns about them, respectively) and 2 scales measure beliefs that, in general, medicines are harmful or overused (range, 4-20; higher score indicates more belief that medicines are harmful or overused).
[d] Assessed using the Diabetes Self-efficacy Scale (range, 8-80; higher score indicates greater feelings of diabetes self-efficacy).
[e] Assessed using the Problem Areas in Diabetes Scale-5 (range, 0-20; a score ≥8 indicates high diabetes distress).
[f] Assessed using the Patient Health Questionnaire-8 (range, 0-20; score ≥10 indicates moderate--to-severe depressive symptoms).

© 2024 Trief PM et al. *JAMA Network Open.*

[g] Assessed using the Generalized Anxiety Disorders Questionnaire-7 (range, 0-21; score ≥10 indicates moderate-to-severe anxiety symptoms).
[h] Assessed using the Chronic Illness Resources Survey (range, 1-5; higher score indicates greater use of support for diabetes self-management).
[i] Assessed using the Material Needs Insecurities Survey. Comparisons are among participants reporting the insecurity vs those not reporting the insecurity.

© 2024 Trief PM et al. *JAMA Network Open.*

**eTable 5.** Psychosocial Factors Associated With $HbA_{1c}$ Level Change Over Time

| Psychosocial Factor | Estimates | S.E. | p-value* |
|---|---|---|---|
| **Psychological Factors** | | | |
| Diabetes attitudes, per 1-point increase[b] | | | |
| Seriousness of diabetes | -0.07 | 0.18 | 0.69 |
| Psychosocial impact | -0.07 | 0.16 | 0.67 |
| Patient autonomy | 0.10 | 0.18 | 0.59 |
| Beliefs about medicines, per 5-point increase[c] | | | |
| Specific beliefs | | | |
| Necessity | -0.07 | 0.05 | 0.18 |
| Concerns | -0.07 | 0.06 | 0.25 |
| General beliefs | | | |
| Harm | 0.03 | 0.15 | 0.84 |
| Overuse | 0.14 | 0.15 | 0.35 |
| Diabetes self-efficacy, per 5-point increase [d] | 0.002 | 0.03 | 0.95 |
| Diabetes distress[e] | | | |
| Per 1-point increase | -0.01 | 0.02 | 0.75 |
| Score ≥8 (vs score <8) | -0.02 | 0.22 | 0.93 |
| Depression symptoms[f] | | | |
| Moderate-to-severe (vs None-to-mild symptoms) | 0.30 | 0.32 | 0.35 |
| Anxiety symptoms[g] | | | |
| Moderate-to-severe (vs None-to-mild symptoms) | -0.01 | 0.40 | 0.99 |
| **Social Factors** | | | |
| Self-management support, per 1-point increase[h] | -0.13 | 0.13 | 0.31 |
| Material Need Insecurity[i] | | | |
| Medication | 0.31 | 0.29 | 0.28 |
| Food | -0.12 | 0.20 | 0.55 |
| Housing | 0.12 | 0.21 | 0.56 |
| Healthcare coverage | 0.17 | 0.27 | 0.53 |
| ≥1 insecurity | 0.17 | 0.24 | 0.47 |
| ≥2 insecurities | 0.10 | 0.23 | 0.65 |

[a] Of 348 participants, 108 (31%) had decreased HbA1c by at least 0.5%, 129 (37%) had increased HbA1c by at least 0.5%, and 111 (32%) had relatively stable HbA1c over time. All generalized linear models were adjusted for baseline BMI and no. of comorbidities/complications.
[b] Assessed using the Diabetes Attitudes Scale (subscale range, 1-5; higher scores indicate greater belief that diabetes is a serious disease, that diabetes has had a greater impact on quality of life, and that persons with diabetes have a right to decide how hard they will work to control their blood glucose).
[c] Assessed using the Beliefs About Medicines Questionnaire: 2 scales measure beliefs in the necessity of ,and concerns about, diabetes medicines (range, 5-25; higher scores indicate more belief that diabetes medicines are necessary and more concerns about them, respectively) and 2 scales measure beliefs that, in general, medicines are harmful or overused (range, 4-20; higher score indicates more belief that medicines are harmful or overused).
[d] Assessed using the Diabetes Self-Efficacy Scale (range, 8-80; higher score indicates greater feelings of diabetes self-efficacy).
[e] Assessed using the Problem Areas in Diabetes Scale-5 (range, 0-20; a score ≥8 indicates high diabetes distress).
[f] Assessed using the Patient Health Questionnaire-8 (range, 0-20; score ≥10 indicates moderate-to-severe depressive symptoms).
[g] Assessed using the Generalized Anxiety Disorders Questionnaire-7 (range, 0-21; score ≥10 indicates moderate--to-severe anxiety symptoms).

© 2024 Trief PM et al. *JAMA Network Open.*

[h] Assessed using the Chronic Illness Resources Survey (range, 1-5; higher score indicates greater use of support for diabetes self-management).
[i] Assessed using the Material Needs Insecurities Survey. Comparisons are among participants reporting the insecurity vs those not reporting the insecurity.

© 2024 Trief PM et al. *JAMA Network Open.*

**eAppendix.** Material Needs Insecurities Survey

**Experiences with Prescription Medications:**

*The following questions concern the use of prescription medication*

1. DURING THE PAST 12 MONTHS, has your doctor prescribed any medication for any condition (Y/N) (If 'No', skip items 2 – 5)

2. DURING THE PAST 12 MONTHS, was there any time when you needed prescription medicines but didn't get it because you couldn't afford it? (Y/N)

3. DURING THE PAST 12 MONTHS, did you skip medication doses to save money? Y/N

4. DURING THE PAST 12 MONTHS, did you take less medicine to save money? Y/N

5. DURING THE PAST 12 MONTHS, did you delay filling your prescription to save money? Y/N

**Experiences with Food:**

***The following questions are about the food eaten in your household in the last 12 months, and whether you were able to afford the food you need.*** *Please read the following statements that people have made about their food situation. For these statements, please mark whether the statement is often true, sometimes true, or never true for you or your household in the last 12 months, that is, for the past year.*

6. The food that I/we bought just didn't last, and I/we didn't have money to get more.

[1= often true, 2= sometimes true, 3= never true]

7. I/we couldn't afford to eat balanced meals.

[1= often true, 2= sometimes true, 3= never true]

8. In the last 12 months, did you or other adults in your household ever cut the size of your meals or skip meals because there wasn't enough money for food? Y/N (If 'No', skip to item 9 )

   a) You indicated you cut the size of your meals or skipped meals because there wasn't enough money for food. How often did this happen?

[1= almost every month; 2= some months but not every month; 3= only 1 or 2 months]

9. In the last 12 months, did you ever eat less than you felt you should because there wasn't enough money for food? Y/N

10. In the last 12 months, were you ever hungry but didn't eat because there wasn't enough money for food? Y/N

© 2024 Trief PM et al. *JAMA Network Open.*

**Experiences with Housing:**

*The following questions ask about your housing.*

11. Do you live in …

1. An apartment
2. A house/townhouse/condo
3. A shelter, transitional living situation (Skip to item 13)
4. Other____________(Specify)
5. Residential treatment/supervised housing
6. Government housing (e.g., army)
7. Mobile home/trailer
8. Room/rented room (Skip to item 13)
9. Car (Skip to item 13)
10. No steady place to sleep at night (Skip to item 13)
11. Hotel/motel (Skip to item 13)
12. Don't know or Don't wish to answer

12. Do you own your own home? In other words, could you sell the home if you wanted to? Y/N/Don't know or Don't wish to answer

13. In the past 3 years, how many places, including your current place, have you lived for one week or longer? [record number; if answer is 1, skip to item 15)

14. Did you ever move because you could no longer afford a home that you were living in? Y/N

15. Have you moved in with anyone in the last 12 months to share household expenses? Y/N

16. Have you ever been homeless at any time in the last 12 months? Y/N

© 2024 Trief PM et al. *JAMA Network Open.*
