# Supplementary material for: Psychosocial Factors and Glycemic Control in Young Adults With Youth-Onset Type 2 Diabetes
Source: JAMA Netw Open. 2024 Apr 8;7(4):e245620. doi: 10.1001/jamanetworkopen.2024.5620 (PMC11002701; doi:10.1001/jamanetworkopen.2024.5620)
Supplement: Supplement 2. — Data Sharing Statement [file jamanetwopen-e245620-s002.pdf]

Data Sharing Statement

Trief. Psychosocial Factors and Glycemic Control in Young Adults With Youth-Onset Type 2 Diabetes. *JAMA Netw Open*. Published April 08, 2024.
doi:10.1001/jamanetworkopen.2024.5620

Data
Data available: Yes
Data types: Deidentified participant data, Data dictionary
How to access data: Data and the data dictionary will be made available upon reasonable request of the corresponding author at triefp@upstate.edu.
When available: With publication

Supporting Documents
Document types: None

Additional Information
Who can access the data: Data will be made available to researchers whose proposed use of the data has been approved.
Types of analyses: Data will be made available for analyses that have pre-specified relevant hypotheses.
Mechanisms of data availability: Data will be made available after a proposal has been approved and with a signed data access agreement.
Any additional restrictions: No additional restrictions.
